# Cervical non-fusion using biomimetic artificial disc and vertebra complex: technical innovation and biomechanics analysis

**DOI:** 10.1186/s13018-022-03012-9

**Published:** 2022-02-23

**Authors:** Jialiang Li, Pengrong OuYang, Xijing He, Xinyu Wei, Zhongwei Sun, Hui Dong, Zhijing Wen, Yibin Wang, Pengzhen Gu, Teng Lu, Ning Liu, Haopeng Li

**Affiliations:** 1 Department of Orthopedics, The Second Affiliated Hospital of Xi’an Jiaotong University, Xi’an, Shaanxi Province China; 2 Department of Health Management, The Second Affiliated Hospital of Xi’an Jiaotong University, Xi’an, Shaanxi Province China; 3 Department of Engineering Mechanics, School of Civil Engineering, Southeast University, Nanjing, China; 4 Department of Spine Surgery, Hanzhong Central Hospital, Hanzhong, Shaanxi Province China

**Keywords:** Biomechanics, Finite element analysis, Cervical vertebrae, Adjacent segment disease, Range of motion, Prostheses and implants

## Abstract

**Background:**

Changes in spinal mobility after vertebral fusion are important factors contributing to adjacent vertebral disease (ASD). As an implant for spinal non-fusion, the motion-preserving prosthesis is an effective method to reduce the incidence of ASD, but its deficiencies hamper the application in clinical. This study designs a novel motion-preserving artificial cervical disc and vertebra complex with an anti-dislocation mechanism (MACDVC-AM) and verifies its effect on the cervical spine.

**Methods:**

The MACDVC-AM was designed on the data of healthy volunteers. The finite element intact model, fusion model, and MACDVC-AM model were constructed, and the range of motion (ROM) and stress of adjacent discs were compared. The biomechanical tests were performed on fifteen cervical specimens, and the stability index ROM (SI-ROM) were calculated.

**Results:**

Compared with the intervertebral ROMs of the intact model, the MACDVC-AM model reduced by 28–70% in adjacent segments and increased by 26–54% in operated segments, but the fusion model showed the opposite result. In contrast to the fusion model, the MACDVC-AM model diminished the stress of adjacent intervertebral discs. In biomechanical tests, the MACDVC-AM group showed no significant difference with the ROMs of the intact group (*p* > 0.05). The SI-ROM of the MACDVC-AM group is negative but close to zero and showed no significant difference with the intact group (*p* > 0.05).

**Conclusions:**

The MACDVC-AM was successfully designed. The results indicate that the MACDVC-AM can provide physiological mobility and stability, reduce adjacent intervertebral compensatory motion, and alleviate the stress change of adjacent discs, which contributes to protect adjacent discs and reduce the occurrence of ASD.

## Background

Cervical fusion surgery, such as anterior cervical corpectomy and fusion (ACCF), are conventional surgical treatments for cervical spondylotic myelopathy (CSM), vertebral trauma, and spinal tumor [1–4]. This type of surgery provides sufficient support that restores the spine stability. However, it inevitably reduces the mobility of the operated segment and increases the stress at the adjacent intervertebral disc (IVD) [5, 6]. As a result, the incidence of adjacent segment disease (ASD) after vertebral fusion is higher [7, 8].

Non-fusion fixation of the cervical spine has become one of the possible ways to solve the ASD in recent years. Motion-preserving treatment is an attractive option especially in the surgical therapy of long-column spinal pathologies as it reduces the incidence of ASD due to the provision not only of excellent stability but also of considerable physiological mobility of the spine. Robertson and Zigler found that the retention of cervical mobility effectively prevented ASD-related imaging changes, caused fewer symptoms, and had a low reoperation rate [9, 10].

However, the traditional motion-preserving spinal prostheses have certain shortcomings in their design. The contact surface of these devices with the vertebral endplate is too flat, which induces a mismatch between the prosthesis and the endplate [11]. Moreover, such devices do not provide a sufficient bone grafting space, which may prolong the process of fusion between the prosthesis and the surrounding bone [12]. In addition, the fixation design of some of these devices have is unsuitable. Nevertheless, these traditional devices lack the capability of an anti-dislocation mechanism, which may cause the articular ball to dislocate from the trough when the cervical spine is subjected to excessive extension and flexion position. These deficiencies have significantly hampered the widespread application of motion-preserving prostheses.

In this study, we designed a novel motion-preserving artificial cervical disc and vertebra complex with an anti-dislocation mechanism (MACDVC-AM), based on the cervical data of healthy volunteers. Then, we compared the biomechanics of the MACDVC-AM with that of vertebral fusion, by using the finite element (FE) method and subjecting the human cervical specimen to a biomechanical test. In the present investigation, we aimed to address and overcome the aforementioned hindrances, which would contribute to the wider application and further development of motion-preserving prostheses.

## Methods

### Measurement of human cervical spine

**Fig. 1:**
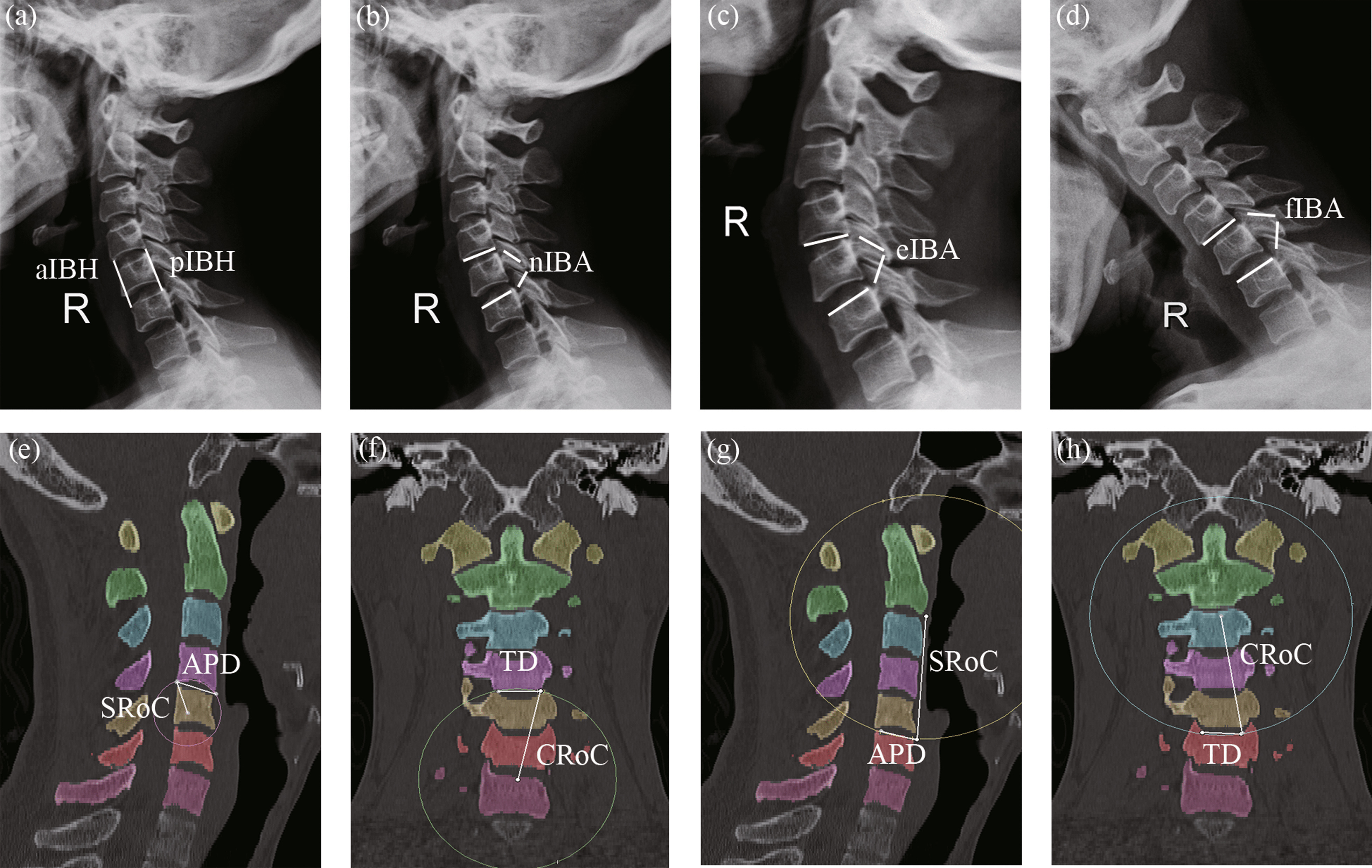
Measurement of parameters of C4–6 in the X-ray and CT images. **a** Measurement of anterior intervertebral body height (aIBH) and posterior intervertebral body height (pIBH); **b** measurement of lateral neutral intervertebral body angle (nIBA); **c** measurement of extension IBA (eIBA); **d** measurement of flexion IB A (fIBA); **e** Measurement of sagittal anteroposterior diameter (APD) and the middle sagittal radius of curvature (SRoC) of C4 lower endplate; **f** Measurement of coronal transverse diameter (TD) and the middle coronal radius of curvature (CRoC) of C4 lower endplate; **g** Measurement of APD and SRoC of C6 upper endplate; **h** Measurement of TD and CRoC of C6 upper endplate

Fifty volunteers (age: 42.37 ± 14.98 years old; 31 females and 19 males) were included in this study. The medical history of each volunteer was reviewed to exclude diseases such as vertebrae deformity, trauma, tumors, and osteoporosis. All volunteers were performed X-ray (QDR-2000; Hologic, Waltham, MA) and CT (SOMATOM Definition AS; SIEMENS, Berlin, Germany). The following parameters were measured in X-ray film (Fig. 1a–d): anterior intervertebral body height (aIBH), posterior intervertebral body height (pIBH), and intervertebral body angle (IBA) in different positions. The CT data were reconstructed to measure the parameters of the endplate geometries of C4 and C6, including the middle sagittal radius of curvature (SRoC) and anteroposterior diameter (APD), and the middle coronal radius of curvature (CRoC) and transverse diameter (TD), respectively (Fig. 1e–h).

### Design of the MACDVC-AM

**Fig. 2:**
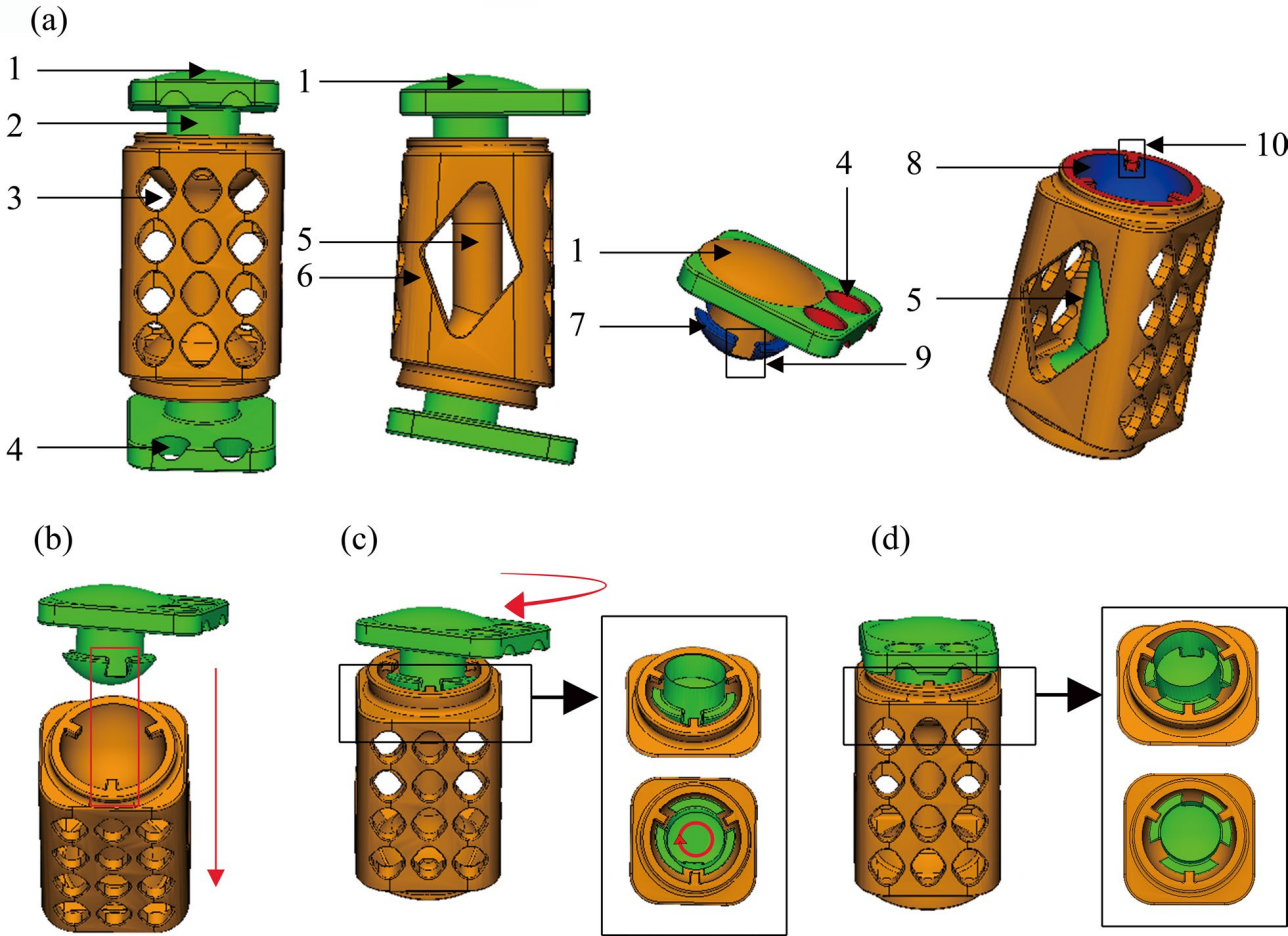
Design of the MACDVC-AM. **a** Front view and lateral view of the MACDVC-AM. 1: supporting structure; 2: handle structure; 3: small windows; 4: nail channels; 5: an internal support structure; 6: windows for inserting bone grafts; 7: artificial articular ball; 8: articular trough structures; 9: three grooves on the artificial articular ball; 10: the three blocks on the articular trough structure. The artificial articular ball (7) and articular trough structures (8) constitute a hemispherical socket joint (ball-in-trough structure); three grooves (9) and three blocks (10) constitute the anti-dislocation mechanism. **b**–**d** The assembling process of the MACDVC-AM. Firstly, assembling the blocks (10) along the grooves (9); Secondly, rotating 60° to reach the standard position so that the articular ball is wholly embedded in the articular trough

The APD, TD, aIBH, pIBH, SRoC, and CRoC were measured to determine the whole size and the geometry of the endplate of the MACDVC-AM. The MACDVC-AM consists of three parts: an upper artificial disc, a lower artificial disc, and an intermediate artificial vertebra (Fig. 2).

**Fig. 3:**
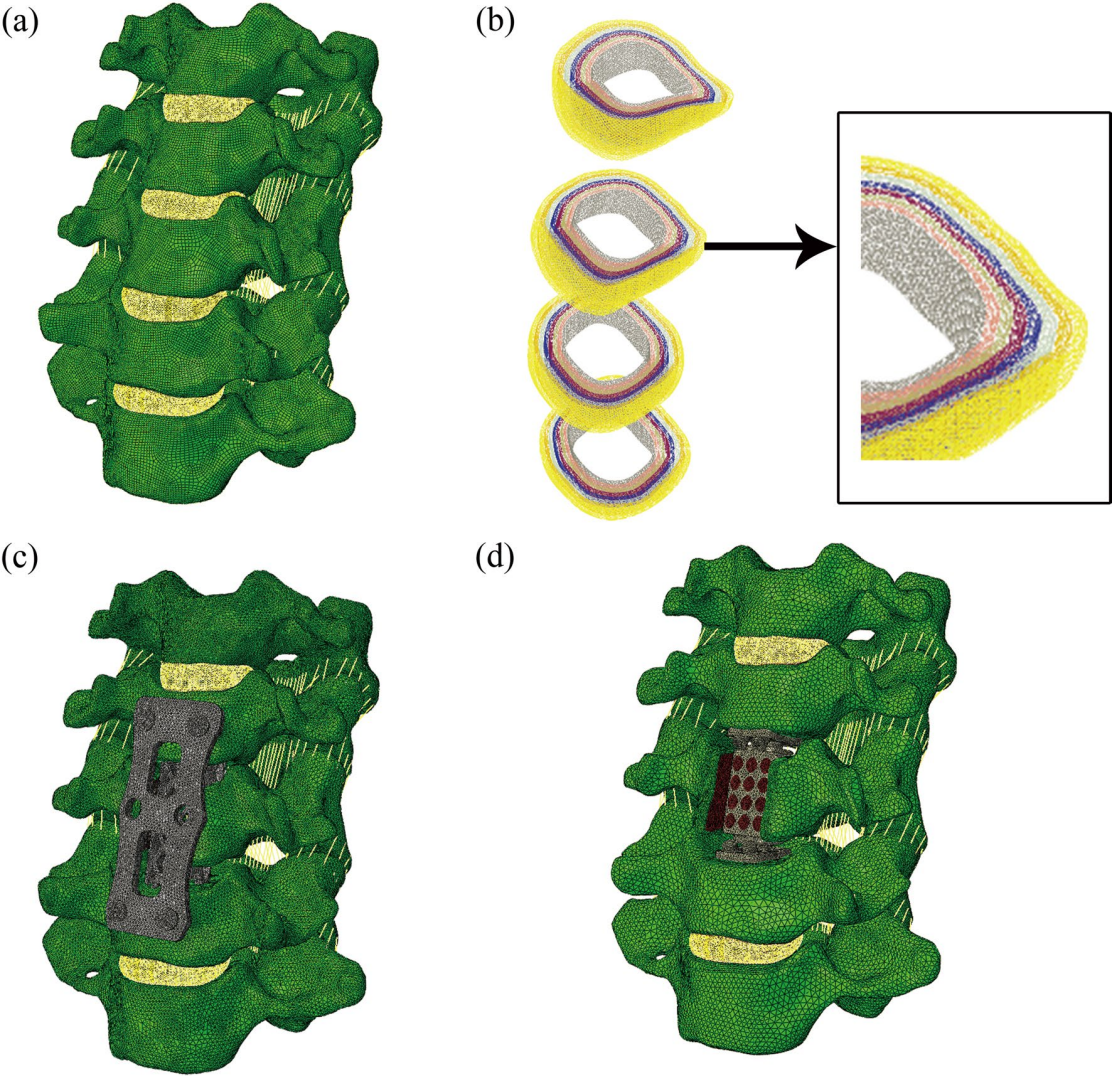
Finite element model of C3–7 cervical spine. **a** Intact model; **b** Annulus fibers; **c** ACCF model; **d** MACDVC-AM model

**Fig. 4:**
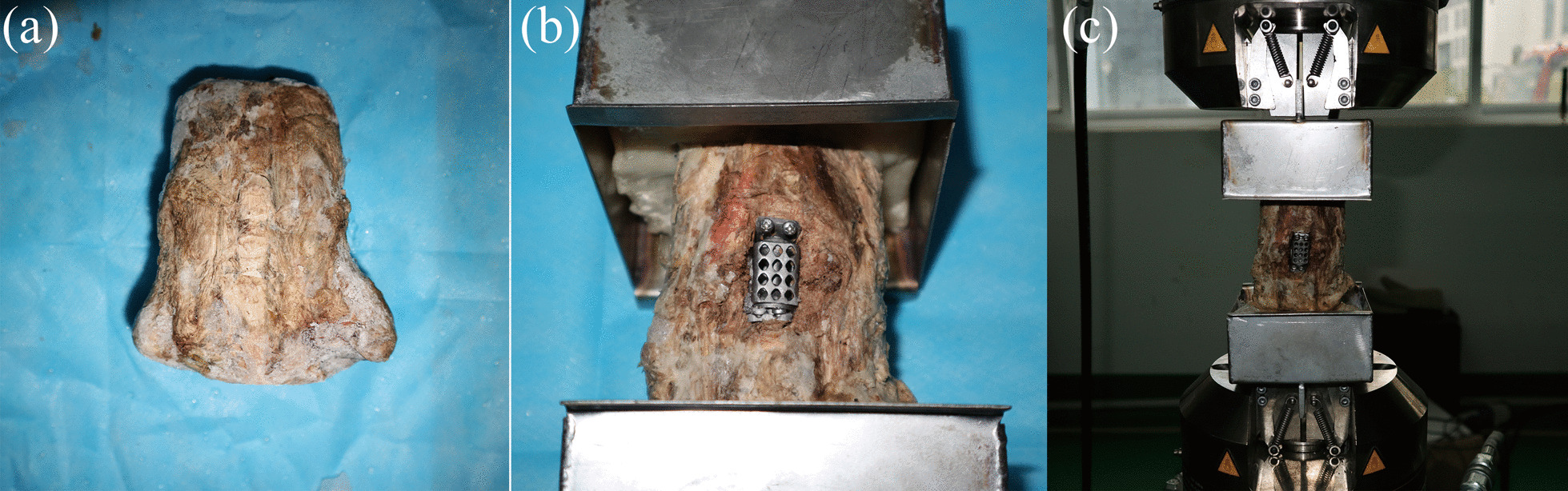
In vitro the MACDVC-AM implantation in human cervical spines. **a** human cervical vertebra specimen; **b** mechanical test model of the MACDVC-AM; **c** biomechanical testing of the MACDVC-AM implanted C4–6 segments using an MTS machine

**Fig. 5:**
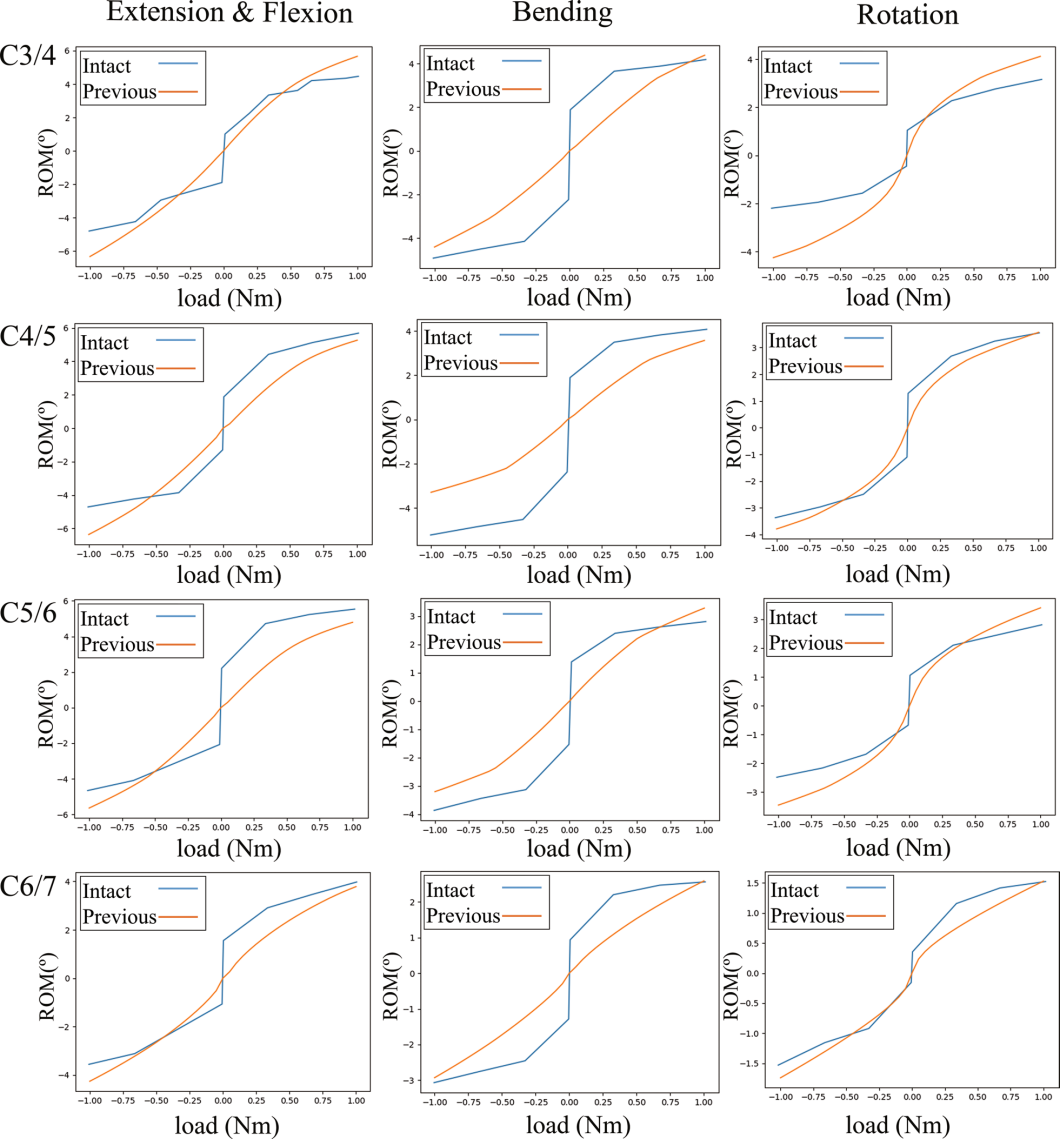
Comparison of the loading process between the FE models. Intact: intact model; Previous: Panjabi study

**Fig. 6:**
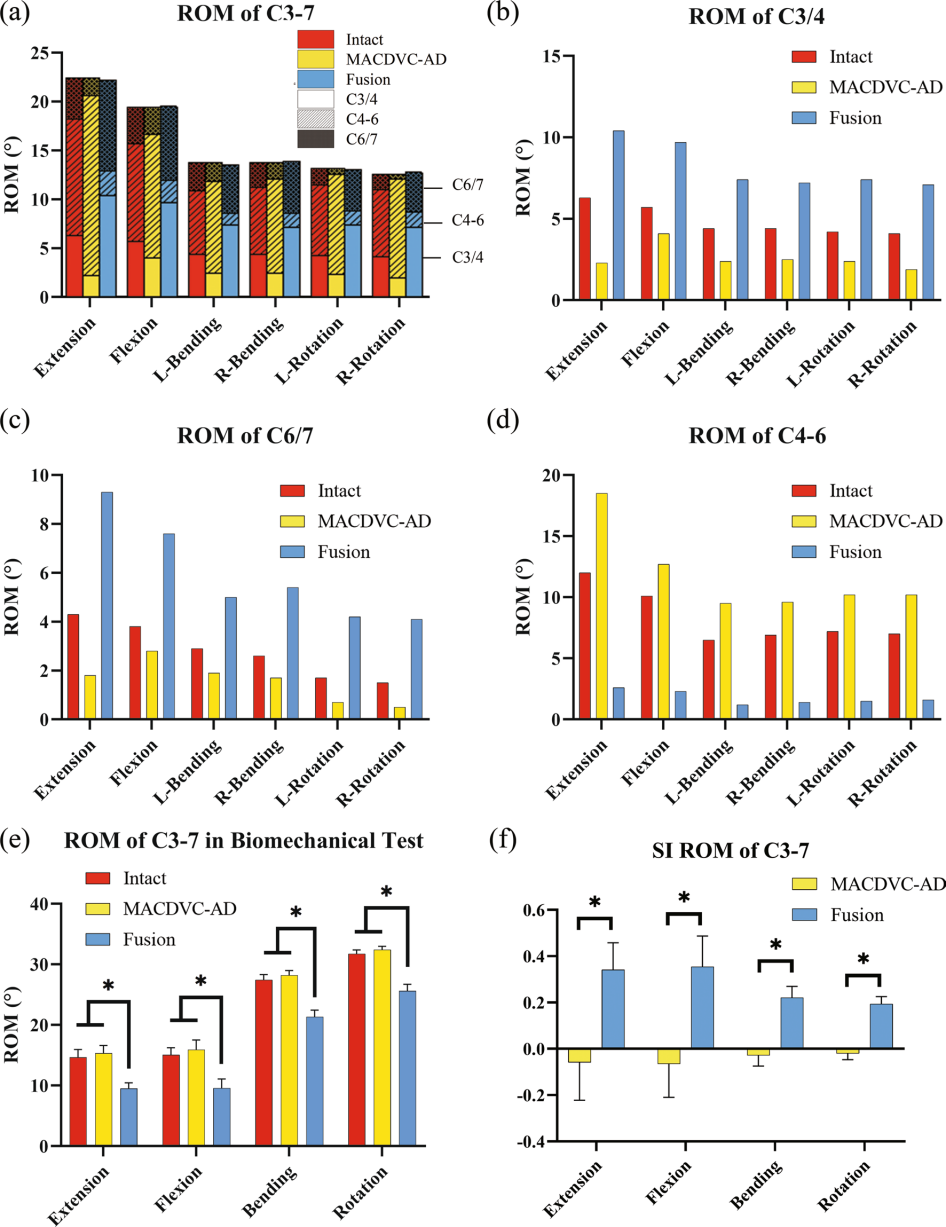
Comparison of ROMs and SI-ROM in FE analysis and biomechanical tests. **a** The contribution of intervertebral ROMs to the overall ROMs of C3–7 in FE models. The bottom of each column is the intervertebral ROMs of C3/4. The middle part of each column, filled by the dotted line, represents the ROMs of C4–6. And the upper part of each column, filled by the dotted line, represents the ROMs of C6/7. The contribution of each intervertebral ROM to the overall ROMs can be seen directly. **b** ROMs of C3/4; **c** ROMs of C6/7; **d** ROMs of C4–6; **e** overall ROMs of C3–7 in the biomechanical test; **f** SI-ROM of C3–7 in the biomechanical test. L-Bending: Left Bending; R-Bending: Right Bending; L-Rotation: Left Rotation; R-Rotation: Right Rotation. SI-ROM: the stability index ROM. Error bar represents 1 SD. *P < 0.05

**Fig. 7:**
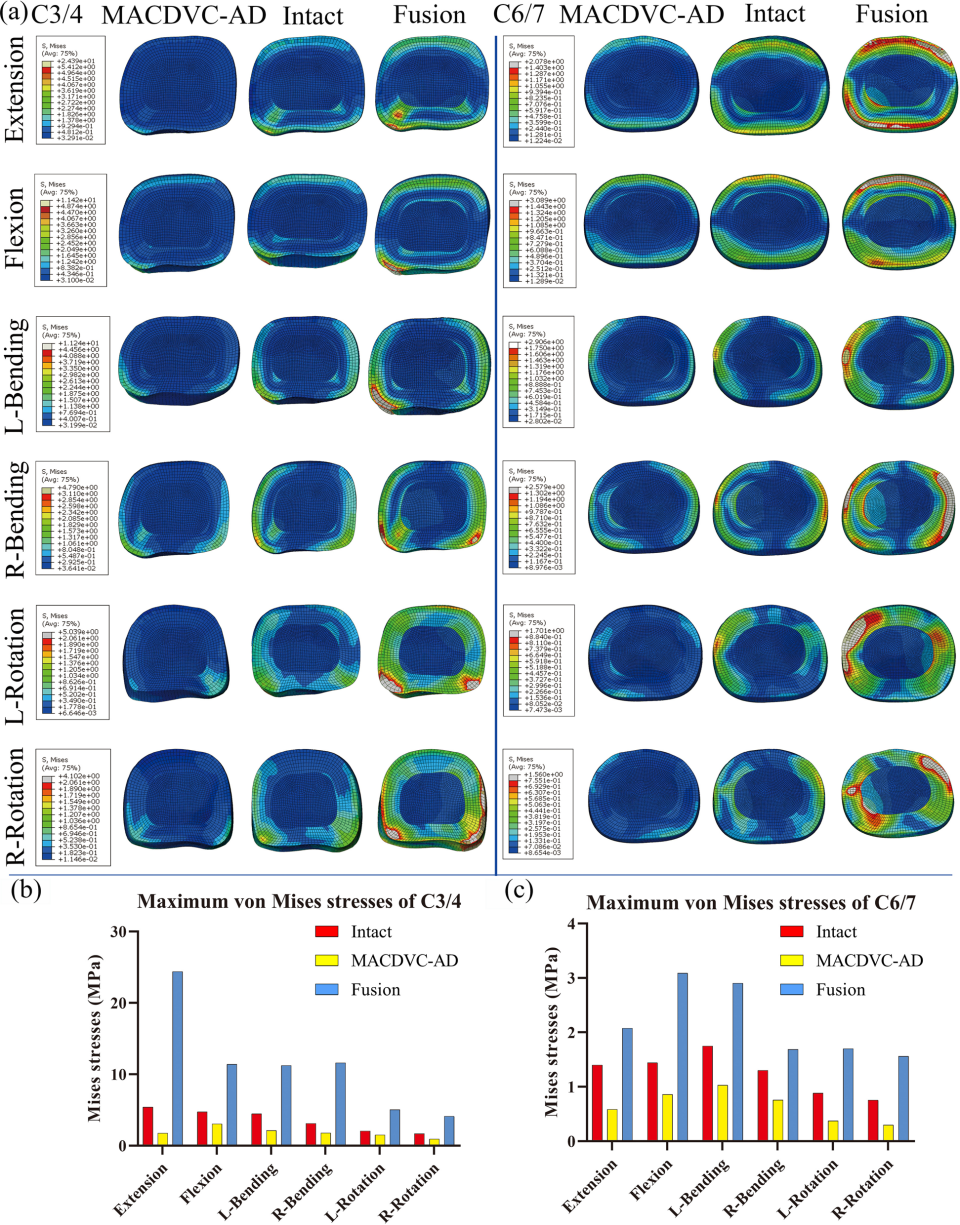
Stress distribution and comparison of the adjacent intervertebral disc. **a** Stress distribution in C3/4 and C6/7; **b** the maximum von Mises stresses on the adjacent intervertebral disc in C3/4; **c** the maximum von Mises stresses on the adjacent intervertebral disc in C6/7

Each artificial disc has four parts, including a supporting structure (Fig. 2a-1), two nail channels (Fig. 2a-4), an artificial articular ball (Fig. 2a-7), and a handle structure (Fig. 2a-2). Due to the lower endplate of the C4 had an arched shape, a dome-shaped supporting structure of the upper artificial disc was designed, moreover, the supporting structure of the lower artificial disc was designed as a flat supporting structure. Two nail channels located in the first quarter of the plate allowing cortical screws with a diameter of 3.5 mm to fix the endplates.

The intermediate artificial vertebra includes articular trough structures on its upper and lower sides (Fig. 2a-8), an internal support structure (Fig. 2a-5), and windows on the lateral side (Fig. 2a-6) for inserting bone grafts. The anterior and posterior small holes (Fig. 2a-3) are designed to reduce the weight of the prosthesis and increase the fusion with the surrounding bone. The oblique angle of the intermediate artificial vertebra was designed to be 10°.

The articular ball and articular trough structures constitute a hemispherical socket joint allowing a ROM of 15° in flexion, extension, and lateral bending, a ROM of 360° in axial rotation, and a 1-mm within a horizontal anterior–posterior slide. The three grooves (Fig. 2a-9) on the artificial articular ball and the three blocks (Fig. 2a-10) on the articular trough structure constitute a unique anti-dislocation mechanism. The blocking function of this anti-dislocation mechanism is as follows. The blocks need to be assembled along the grooves and rotated by 60° to reach the standard position so that the articular ball is wholly embedded in the trough structures. The limitations imposed by the anti-dislocation mechanism prevent the escape of the articular ball from the articular trough in all directions.

### Finite element analysis

#### Finite element model of C3–7

A nonlinear FE model was constructed in the following steps. The CT data of a healthy volunteer (55-year-old, male, 175 cm, 75 kg) were imported to the MIMICS (Materialise Inc., Leuven, Belgium) to reconstruct the bone tissue. Then geometric models of the cortical shell, cancellous bone, and IVDs were developed in 3-Matic (Materialise Inc., Leuven, Belgium). Then, mesh models were designed, and material property assignment was conducted in Hypermesh (Altair Engineering, Inc., Troy, MI, USA). Finally, ABAQUS (Hibbitt, Karlsson and Sorenson, Inc., Providence, RI, USA) was used for FE analysis.

The C3–7 intact FE model, including five vertebrae, four IVDs, the anterior and posterior longitudinal ligament, the ligamentum flavum, the interspinous ligament, and the capsular ligament (Fig. 3a). The thickness and the area of articular cartilage were 0.5 mm and 80–100 mm^2^, respectively [13–15]. In the IVDs, the volume of the nucleus pulposus accounted for approximately 40%; the nucleus pulposus and annulus grounds were defined as incompressible hyperelastic fluid using a Moony–Rivlin model [16]. The angle between the annulus fibrous and the cartilage endplate was 25°–35°, and the annulus fibrous contained eight layers of collagen fibers (Fig. 3b) [17, 18]. A nonlinear hypoelastic constitutive relationship was utilized to approximate the ligament, based on the results of our previous study [19]. The material properties used in the FE model are listed in Table 1 [16, 17, 20–22].

**Table 1:** Material properties assigned to the finite element models

Component	Element type	Number of element	Young modulus (Mpa)	Poisson ratio	Cross-sectional area (mm^2^)
Cortical bone	C3D8	205,740	10,000	0.3	
Cortical endplate	C3D4	36,902	3000	0.25	
Cancellous bone	C3D4	1,274,162	100	0.2	
Cartilaginous endplate	C3D8	16,002	24	0.4	
Nucleus pulpous	C3D8	68,229	Hyperelastic *C*_10_ = 0.12 *C*_01_ = 0.09		
Annulus grounds	C3D8	44,037	Hyperelastic *C*_10_ = 0.56 *C*_01_ = 0.14		
Annulus fibers	T3D2	1,762,759	Nonlinear^a^	0.3	
Anterior longitudinal	T3D2	574	Nonlinear^a^	0.3	11.1
Posterior longitudinal	T3D2	72,096	Nonlinear^a^	0.3	11.3
Ligamentum flavum	T3D2	73	Nonlinear^a^	0.3	46.0
Capsular ligament	T3D2	123	Nonlinear^a^	0.3	42.2
Interspinous	T3D2	40	Nonlinear^a^	0.3	13.0
Titanium alloy	C3D4	–	110,000	0.3	

^a^Ligament properties are referred to a previous study (Liu et al. 2019) [19] for details

#### Finite element model of fusion and MACDVC-AM

In fusion model, to simulate the ACCF operation, two-thirds of the vertebrae of C5, the IVDs of C4–6, and the anterior and posterior longitudinal ligaments were removed. A titanium mesh cage (TMC) was implanted and fixed by an anterior plate-screw system (Fig. 3c). The MACDVC-AM model (Fig. 3d) was constructed as follows. Similar to the ACCF operation, the patient was in the supine position and underwent a subtotal corpectomy of the C5. Then, the MACDVC-AM was assembled and implanted into the operated area. Next, the surfaces of MACDVC-AM were assured to fit the endplates, and the upper and lower artificial discs were fixed by two screws to the endplates of C4 and C6, respectively. For the fusion and MACDVC-AM model, the interfaces at the implant-endplate and screw-bone were defined as a tied contact condition to simulate a complete fusion status [23]. Finally, a convergence analysis in vertebral and disc meshes was conducted to ensure that the maximum changes in the strain energy were less than 5% [24]. The mesh element numbers are presented in Table 1.

#### Loading and boundary conditions

For all FE models, we constrained the C7 lower endplate in all six directions and set a reference point on the C3 upper endplate. An axial preload of 73.6 N was applied to simulate physiological compression [25]. A 1-Nm moment load was then employed to produce flexion, extension, lateral bending, and axial rotation. The validity of the current FE model was demonstrated by comparing the intervertebral ranges of motion (ROM) of the intact model with those of previous studies [26–29]. To reduce the influence of ROMs and other index changes, caused by different prostheses, the overall ROMs of the experimental model and control model had to be consistent [15, 19, 25]. Hence, a displacement-control loading that equaled that of the intact model was applied to the ACCF and MACDVC-AM model to compare the intervertebral ROMs. The Mises stress values of the adjacent disc nucleus pulposus (C3/4 and C6/7) were obtained simultaneously.

### Biomechanical tests

#### Specimens

Fifteen human cervical specimens (C2-T1; age: 57.80 ± 6.39 years; 7 males and 8 females) were harvested from the Anatomy Department of Xi'an Jiaotong University. X-ray examinations were performed to exclude deformity, osteoporosis, and degeneration. The muscles and soft tissues around the vertebra were carefully removed, but the small joints, ligaments, and bony structures were preserved (Fig. 4a). The specimens were stored at -20 ℃ ﻿in plastic bags.

#### Biomechanical models

All TMC and MACDVC-AM were manufactured from Ti6Al4V alloy using a selective laser melting 3D-printing machine (BLT-S300, Bright Laser Technologies, Xi'an, China). Fifteen specimens were randomly divided into three groups: intact, fusion (with TMC and an anterior plate-screw system), and MACDVC-AM groups. After TMC and MACDVC-AM implantation, each specimen was subjected to an X-ray examination for determination of the position of the implant, followed by a fixation on a polymethyl methacrylate matrix to provide stability during the biomechanical test (Fig. 4b).

#### Three-dimensional mobility tests

An MTS rotating machine (MTS-858/2.5, MTS System Inc., Minneapolis, MN, USA) was used in the biomechanical tests for the evaluation of specimen mobility (Fig. 4c). The specimens were embedded in a particular metal mold with the C3 vertebra parallel to the horizontal plane. A 2-Nm moment load was applied to produce flexion, extension, lateral bending, and axial rotation [30, 31]. The axial rotation ROMs of the C3–7 was directly recorded by a computer. Using previously reported methods [32], we marked the C3 and C7 and recorded their locations by fixed cameras at zero and maximal loads. The markers in the images were identified by an image processing system, and the movement angles were determined as the flexion, extension, lateral bending ROMs. Before the experiment, all specimens were incubated at room temperature. During the test, saline solution was employed to maintain the moisture of the specimens.

#### Stability index flexibility test

The stability index ROM (SI-ROM) is utilized to quantify the stability that prostheses provide to specimens [30, 33]. The SI-ROM is defined by the following formula: SI-ROM = (ROMintact − ROMprosthesis)/ROMintact. An SI-ROM value of zero indicates that the stability of the test model is equal to that of the intact model. A positive value shows that the test model is more stable than the intact model. In contrast, a negative value shows that the test model is less stable than the intact model.

### Statistical analysis

The data were analyzed by SPSS (version 19.0, SPSS Inc., Chicago, IL, USA). Data are expressed as mean ± SD. A repeated-measures analysis of variance (Brown–Forsythe and Welch ANOVA tests) with Tukey *post-hoc* tests was conducted to compare intervertebral ROMs and the maximum von Mises stresses, and a two-tailed Student's *t*-test was conducted to compare the SI-ROM. *p* values < 0.05 were considered to indicate statistically significant differences.

## Results

### Imaging data of human cervical spine

As can be observed in Table 2, the C5 vertebra was wedge-shaped with high aIBH (23.24 ± 2.14 mm) and low pIBH (20.71 ± 1.73 mm). The lower C4 endplate was dome-shaped with a low SRoC value (21.56 ± 8.07 mm). The SRoC (115.25 ± 69.29 mm) and CRoC (95.18 ± 59.10 mm) values of the upper C6 endplate were high, and thus the upper C6 endplate was regarded as a platform. The APD values of the endplates (16.63 ± 2.13 mm in the lower C4 and 16.50 ± 2.21 mm in the upper C6) were almost equal. The TD of the endplates (16.97 ± 1.82 mm in the lower C4 and 16.90 ± 2 0.49 mm in the upper C6) were the same.

**Table 2:** Digital X-ray and CT data of the C4–6

Cervical vertebrae	IBH (mm)	aIBH	23.24 ± 2.14
		pIBH	20.71 ± 1.73
	IBA (°)	nIBA	10.18 ± 2.44
		eIBA	17.85 ± 3.51
		fIBA	2.50 ± 3.39
		rIBA	15.35 ± 4.77
Endplate	Lower C4 (mm)	APD	16.63 ± 2.13
		TD	16.97 ± 1.82
		SRoC	21.56 ± 8.07
		CRoC	129.63 ± 53.85
	Upper C6 (mm)	APD	16.50 ± 2.21
		TD	16.90 ± 2.49
		SRoC	115.25 ± 69.29
		CRoC	95.18 ± 59.10

Data are expressed as mean ± SD

aIBH, anterior intervertebral body height; pIBH, posterior intervertebral body height; nIBA, neutral lateral intervertebral body angle; eIBA, extension IBA; fIBA, flexion IBA; APD, anteroposterior diameter; TD, transverse diameter; SRoC, sagittal radius of curvature; CRoC, coronal radius of curvature

### Validation of the intact FE model

The comparison between the intervertebral ROMs of our intact model and those of previous studies is shown in Table 3. Although the intervertebral ROMs of the FE model in this experiment and Panjabi’s study were smaller than those measured in vivo experiments, the intervertebral ROMs of our FE model was equal to that of Panjabi’s FE model under the same loading process (Fig. 5). Therefore, the FE model developed in this study was feasible and valid.

**Table 3:** Comparison of the intersegmental ROMs (°) with those in previous studies

	Current study	Panjabi et al. [27] (2001)	Zhou et al. [28] (2019)	Anderst et al. [29] (2015)
	*In extension and flexion position*			
C3/4	11.99	9.26	14.9 ± 4.0	17.1 ± 3.3
C4/5	11.62	10.40	19.4 ± 2.9	19.5 ± 3.4
C5/6	10.43	10.19	17.1 ± 4.2	19.7 ± 3.7
C6/7	8.05	7.54	12.3 ± 4.2	15.8 ± 4.8
	*In lateral bending position*			
C3/4	8.78	9.098	12.4 ± 3.1	14.3 ± 2.8
C4/5	6.86	9.294	9.8 ± 2.4	13.1 ± 3.2
C5/6	6.49	6.678	10.0 ± 2.3	12.3 ± 3.2
C6/7	5.52	5.629	10.6 ± 3.3	14.5 ± 3.9
	*In axial rotation position*			
C3/4	8.36	5.352	4.2 ± 2.7	11.8 ± 2.1
C4/5	7.35	6.915	6.5 ± 1.9	11.3 ± 1.7
C5/6	6.85	5.295	6.5 ± 2.1	9.3 ± 1.9
C6/7	3.27	3.051	2.4 ± 1.2	6.5 ± 1.7

### ROMs of FE models

The comparison of adjacent intervertebral ROMs, under equal overall ROMs of C3–7, reflects the influence of the fusion and motion-preserving fixation on adjacent IVDs. The contribution of intervertebral ROMs to the overall ROMs under equal displacement-control loading is depicted in Fig. 6a. In the fusion model, the contribution of the intervertebral ROMs of the operated segment to the overall ROM was low, and the adjacent intervertebral ROMs increased, which indicated the action of a compensatory motion of the adjacent IVDs. However, in the MACDVC-AM model, the main motion occurred in the operated segment.

#### ROMs of the adjacent segments: C3/4 and C6/7

The ROMs of C3/4 and C6/7 are illustrated in Fig. 6b, c, respectively. In extension, the ROM of the fusion model was higher than that of the intact model, whereas the one of the MACDVC-AM model was lower by 64% in C3/4 and by 57% in C6/7. In flexion, the MACDVC-AM in C3/4 and C6/7 were both lower by 28%, and the fusion model was higher by 72% in C3/4 and by 101% in C6/7. Moreover, in lateral bending, the MACDVC-AM value in the intact model was lower by 44% in C3/4 and 33% in C6/7, whereas the fusion model was higher values reaching 166–189% in adjacent segments. Furthermore, in axial rotation, the MACDVC-AM dropped by 51% in C3/4 and 70% in C6/7, whereas in the fusion model, the values increased by 172% in C3/4 and by 150% in C6/7.

#### ROMs of operated segment C4–6

Figure 6d shows the ROMs of C4–6. In extension, there was a 54% increase in the MACDVC-AM and a 79% decrease in the fusion model as compared with the intact model. Moreover, in flexion, the MACDVC-AM was 26% higher but lower by 77% in the fusion model. In lateral bending, MACDVC-AM was 43% higher than the intact model, whereas, in the fusion model, it declined by 80%. Furthermore, in axial rotation, the MACDVC-AM was increased by 44% as compared with the intact model, whereas in the fusion model, it dropped by 78%.

### Analysis of the maximum von Mises stress

As can be observed in Fig. 7, the maximum von Mises stress values of the cervical disc nucleus pulposus of the intact model in six directions (in extension, flexion, left bending, right bending, left rotation, and right rotation) in C3/4 were 5.41, 4.74, 4.46, 3.11, 2.06, and 1.69 (MPa), respectively. On the other hand, those of the MACDVC-AM model in each direction were 1.75, 3.05, 2.15, 1.78, 1.50, and 0.95 (MPa), correspondingly. In the fusion model, these values were 24.39, 11.41, 11.24, 11.60, 5.04, and 4.10 (MPa), respectively. The maximum stress values in C6/7in the intact model in six directions were 1.40, 1.44, 1.75, 1.30, 0.88, and 0.76 (MPa), correspondingly; and that of the MACDVC-AM model is 0.58, 0.86, 1.03, 0.76, 0.37, and 0.30 (MPa), respectively; moreover, that of the fusion model is 2.07, 3.09, 2.91, 1.69,1.70, and 1.56 (MPa), respectively. Furthermore, the stress distribution in IVDs indicates the area of increased stress of the fusion model was significantly larger than that of the intact model, and that of the MACDVC-AM model is less than that of the intact model.

### Stability and mobility test

The overall ROMs obtained in the biomechanical test are illustrated in Fig. 6e. The ROMs of the MACDVC-AM group was not statistically significantly different from those of the intact group (*P* > 0.05). However, the ROMs in all directions in the MACDVC-AM group were significantly higher than those in the fusion group (*P* < 0.05). The results of the SI-ROM are presented in Fig. 6f. The SI-ROM of MACDVC-AM is negative but close to zero, whereas the SI-ROM of the fusion group is positive. The SI-ROM values in all directions in the fusion treatment were significantly higher than the ones in the intact treatment (*P* < 0.05). However, the SI-ROM of MACDVC-AM showed no significant difference from that of the intact group (*P* > 0.05).

## Discussion

In recent years, the incidence of vertebral diseases has increased [34, 35]. The pathophysiology of cervical spondylosis and myelopathy causes compression on the nerve roots and spinal cord and induces dysfunction [36–38]. Cervical metastatic tumors and trauma also seriously affect the physiological function of the spine and are even life-threatening. Although, 2-level discectomy is a common method of anterior cervical surgery, subtotal vertebral resection, such as 1-level corpectomy, is required when the ossification of posterior longitudinal ligament, vertebral fracture, vertebral tumor, vertebral hyperplasia, and so on, were observed in the spine. ACCF is used as a conventional treatment of these vertebral diseases, especially of multilevel degenerative cervical stenosis [39]. In the USA, nearly 150,000 patients undergo cervical fusion every year [40]. TMC, which provides sufficient support to rebuild spinal stability, is widely used in ACCF, and good clinical results have been reported [41, 42]. However, cervical fusion inevitably reduces the mobility of the operated level, which increases the compensatory motion of adjacent IVDs [5, 43]. Additionally, the vertebral fusion increases the stress at the adjacent IVDs, inducing upregulation of IL-1 beta and TNF-alpha expression [5, 6, 44]. Besides, the over-compression of the adjacent IVDs inhibits the diffusion of oxygen and nutrients from the endplate leading to disk degeneration [45]. Excessive compensatory motion and stress changes in adjacent IVDs can damage IVDs and induce ASD [7, 8, 46, 47]. Also, damaged adjacent discs can lead to degeneration of other adjacent discs, which accelerates the recurrence of severe CSM [6, 48]. Nevertheless, non-fusion fixation can reduce the compensatory motion and adjacent IVD stress and can reportedly solve the problems of fusion and reduce the incidence of ASD [9–12, 49]. The retention of mobility can reduce the impact of vertebral fusion on the lifestyle activities of patients, such as turning head and driving. However, the anti-dislocation mechanism, fixation, and contact surface morphology of traditional motion-preserving prostheses have not been well designed.

In this study, we successfully designed a MACDVC-AM with improved structures: an anti-dislocation mechanism to avoid the occurrence of dislocation, suitable nail channels to reduce the pressure on the front tissue, and an ample bone graft space to increase long-term stability, etc. Then, by FE analysis and biomechanical test, we established that MACDVC-AM not only maintained the physiological ROMs of the cervical spine but also diminished the stress changes of adjacent IVDs, which might contribute to the protection of adjacent IVDs. Finally, we hope to promote the novel design of motion-preserving prostheses through the successful development of MACDVC-AM and its advantages.

### Details of the MACDVC-AM

APD, TD, aIBH, and pIBH (Table 2) were measured to determine the size of the MACDVC-AM. Based on these data, we designed the MACDVC-AM of different sizes, in which the average front height was 23 mm, the average rear height was 20 mm and the average width was 13 mm. The SRoC and CRoC (Table 2) were used to estimate the geometry of the endplate and for the design of the support structure of the MACDVC-AM. The SRoC of the lower endplate of the C4 is small and has obvious radian, which is the basis for the design of supporting structure in the artificial disc of the MACDVC-AM.

The mismatch between the prostheses and endplate can cause subsidence which induces fixation looseness and reoperation [50, 51]. Previous research found that dome-shaped TMC can significantly reduce the subsidence, and matching with the endplate can also increase the maximum load by 53.8% [52, 53]. In this study, we established that the lower endplate of the C4 had an arched shape, and thus a dome-shaped supporting structure of the upper artificial disc was designed (Fig. 2a-1). This dome-shaped supporting structure can fully contact the lower endplate of the C4 so that the force on the prosthesis and endplate is uniform, and the possibility of prosthesis subsidence is reduced. Moreover, because the upper endplate of C6 was almost flat, the supporting structure of the lower artificial disc was designed as a flat supporting structure.

Traditional prostheses are often fixed with anterior nails and a plate, which causes compression of the trachea and esophagus, as well as dysphagia and even esophageal fistula [54–56]. Lesser anterior fixation can significantly reduce the occurrence of esophageal irritation and dysphagia [57–59]. The nail channels of the MACDVC-AM are located in the first quarter of the plate and at an angle of 40° to the axis, allowing cortical screws with a diameter of 3.5 mm to fix the endplates (Fig. 2a-4). In a previous investigation, Nagaraja found that the mechanical strength which this fixation method provided was similar to that of frontal fixation [60]. MACDVC-AM can be used standalone without cervical plates, thus it potentially mitigates esophageal irritation and dysphagia.

Two large windows for bone grafting were located on the lateral side of the titanium mesh frame, and other small windows were designed to increase the bone contact area (Fig. 2a-6), which facilitated bone fusion. The anterior and posterior small holes (Fig. 2a-3) are designed to reduce the weight of the prosthesis because too much titanium alloy will affect the conduction of electricity, and these small holes also can increase the fusion with the surrounding bone. Based on nIBA (Table 2), the oblique angle of the intermediate artificial vertebra was designed to be 10°. Previously, Lu and Wang proposed that an oblique angle could improve mechanical capacity [52, 53].

The articular ball (Fig. 2a-7) on the artificial disc matches the articular trough structures on the intermediate artificial vertebra (Fig. 2a-8). The articular ball and articular trough structures constitute a hemispherical socket joint (ball-in-trough structure), which allows a ROM of 15° in flexion, extension, and lateral bending, a ROM of 360° in axial rotation. The micro-motion, a 1-mm horizontal anterior–posterior slide within the artificial ball-in-trough structure, should be able to tolerate positional surgery errors, mimicking the physiological motion characteristic of a mobile rotation center. It is worth noting that the micro-motion in traditional artificial implants without an anti-dislocation mechanism may cause a dislocation of the articular ball from the articular trough when the cervical spine is in excessive extension and flexion position. This dislocation can cause serious problems such as severe spinal cord compression and even death.

In this study, we designed a unique anti-dislocation mechanism in the MACDVC-AM: three grooves and three blocks, in Fig. 2 9–10, which are distributed at 120° intervals on the articular ball and articular trough structures, respectively. The assembly of our MACDVC-AM is easy and can be done following these steps (Fig. 2b–d). First, the blocks need to be assembled along the grooves. Then, they are rotated by 60° to reach the standard position so that the articular ball is wholly embedded in the trough structures. The limitations imposed by the anti-dislocation mechanism prevent the escape of the articular ball from the articular trough in all directions. The dislocation occurs only when the hemispherical socket joint is rotated 60° from the standard position, and the three blocks of the articular trough structures are bent and stretched along the side of the three grooves of the articular ball. This situation is difficult to achieve, so the anti-dislocation mechanism is effective to a certain extent.

### Finite element analysis

The loading process of the intact model described here and the results of the previous study [27] are in good agreement (Fig. 5), which indicates the accuracy of the intact model and its potential to be used for subsequent experiments. The contribution of intervertebral ROMs to the overall ROMs is shown in Fig. 6a. The adjacent intervertebral ROMs of the fusion model were larger, whereas the ROMs of the operated segments were significantly lower, meaning that the cervical spine lost a certain degree of its mobility, and a compensatory motion occurs in the adjacent IVDs, which is a major factor for the acceleration of disc degeneration [5–8]. Nonetheless, the MACDVC-AM can provide sufficient mobility in the operated segment, which means that this prosthesis could provide considerable physiological mobility of the spine. This result is similar to those of a previous study [61]. Surprisingly, the increase of ROMs of the C6/7 is significantly higher than that of the C3/4 in the fusion model, so the C6/7 segment might be more prone to degeneration after C4–6 fusion.

Besides, the stress distribution and the maximum stress analysis show the same result (Fig. 7). The stress increase area and the maximum stresses of the fusion model are significantly higher than those of the intact model, which indicates that vertebral fusion can magnify the stress change of the adjacent IVDs, accelerating the degeneration, which explains the occurrence of ASD after ACCF [62]. However, that of the MACDVC-AM model is significantly less than the intact model and the fusion model. The results reveal that the MACDVC-AM has little effect on the stress of the adjacent IVDs. Thus, the preservation of cervical spinal movement can prevent adjacent disc overload [63].

### Biomechanical tests

The prosthesis stability is related to the biomechanical properties of the spine, which can be reflected by the SI-ROM [30]. The SI-ROM of the fusion group was positive and significantly increased as compared with the one of the intact group (*P* < 0.05), which confirms once again that TMC can provide excellent stability [30]. However, the SI-ROM of the MACDVC-AM had a negative value but close to zero, showing no significant difference from that of the intact group (*P* > 0.05), which implies that MACDVC-AM can provide stability similar to that of the intact model (Fig. 6f). Besides, we found that MACDVC-AM ensured the same mobility as the intact model and led to significantly higher overall ROMs than that of the fusion model (Fig. 6e). This result shows that MACDVC-AM can maintain the physiological mobility of the cervical spine. We can, therefore, speculate that MACDVC-AM can reduce the compensatory movement, as compared with that in the vertebral fusion, which could decrease the occurrence of ASD. Above all, the MACDVC-AM not only can improve stability but also has much better performance in providing physiological mobility compared with that of the vertebral fusion.

### Limitation

The limitations of this study must be acknowledged. The morphology of the fixation system and bone graft were simplified to reduce the computation load, and these simplifications inevitably affected the actual stress distribution. Although using the same displacement-control loading can highlight the influence of fusion and motion-preserving fixation on adjacent IVDs, it may ignore the overall situation of the spine. Besides, animal models would be established to test the biomechanics of the prosthesis in future experiments.

## Conclusion

A novel motion-preserving prosthesis was successfully designed in the current study. The MACDVC-AM, with unique structural designs, can provide identical post-operation spinal stability and mobility, and contribute less stress on the adjacent IVDs, which might decrease the occurrence of ASD compared with vertebral fusion in the treatment of cervical diseases.

## Data Availability

Not applicable.

## Abbreviations

ACCF: Anterior cervical corpectomy and fusion
CSM: Cervical spondylotic myelopathy
IVD: Intervertebral disc
ASD: Adjacent segment disease
FE: Finite element
aIBH: Anterior intervertebral body height
pIBH: Posterior intervertebral body height
IBA: Intervertebral body angle
APD: Anteroposterior diameter
TD: Transverse diameter
SRoC: Sagittal radius of curvature
CRoC: Coronal radius of curvature
TMC: Titanium mesh cage
ROM: Range of motion
SI-ROM: Stability index ROM
